# Profiling of Metabolites in Organically Grown Plums from Norway: Does Location or Cultivar Matter?

**DOI:** 10.3390/antiox13050526

**Published:** 2024-04-26

**Authors:** Mekjell Meland, Dragana Dabić Zagorac, Mihajlo Jakanovski, Milica Sredojević, Maja Natić, Marko Kitanović, Milica Fotirić Akšić

**Affiliations:** 1 Norwegian Institute of Bioeconomy Research—NIBIO Ullensvang, Ullensvangvegen 1005, 5781 Lofthus, Norway; 2 Innovative Centre of the Faculty of Chemistry, University of Belgrade, Studentski trg 12–16, 11158 Belgrade, Serbia; ddabic@chem.bg.ac.rs (D.D.Z.); jakanovski@chem.bg.ac.rs (M.J.); pantelicm@chem.bg.ac.rs (M.S.); 3 Faculty of Chemistry, University of Belgrade, Studentski trg 12–16, 11158 Belgrade, Serbia; mnatic@gmail.com; 4 Faculty of Agriculture, University of Belgrade, Nemanjina 6, 11080 Belgrade, Serbia; marko.kitanovic@agrif.bg.ac.rs (M.K.); fotiric@agrif.bg.ac.rs (M.F.A.)

**Keywords:** plum, organic production, metabolites, geographical origin, biological origin

## Abstract

The aim of this work was to investigate the influence of two locations and seven cultivars on the profiling of metabolites in organically grown plums (*Prunus domestica* L.) fruit in Norway. P, K, and Ca were most abundant in the studied fruits, while Ba and Sr formed a clear line between the locations. The most abundant sugars were glucose, fructose, sucrose, and sorbitol, which together accounted for up to 97.00%. Quinic acid and malic acid were the predominant organic acids, while chlorogenic acid, rutin, and kaempferol-3-*O*-glucoside were the most abundant polyphenols. Plums from Ullensvang were characterized by a higher content of minerals, sugars, organic acids, total polyphenol content (TPC), and radical scavenging activity (RSA), while plums from Telemark had a higher content of quantified polyphenols. The cultivar ‘Mallard’ had the highest mineral and radical scavenging activity, ‘Opal’ had the sweetest fruit, ‘Jubileum’ had the highest acidity, ‘Excalibur’ had the highest TPC content, and ‘Valor’ stored the highest content of quantified polyphenols, especially chlorogenic acid. These results provide comprehensive information on the chemical profiles of selected plum cultivars, suggesting that organic plums are a rich source of beneficial compounds that can have a positive impact on human health.

## 1. Introduction

In recent years, intensive traditional production has raised serious concerns about the impact on agricultural sustainability and environmental health [1]. Fruit production has a much higher environmental impact on an area or yield basis than most other crops due to the high application rates of synthetic inputs [2]. One of the solutions to this sensitive environment is organic production. This system puts the principles of health, ecology, fairness, and care together into practice. It relies on renewable energy and ecological principles, promotes resource cycling, improves ecological balance, improves physical and chemical soil characteristics, reduces soil erosion, conserves wildlife and natural habitats, improves animal welfare, reduces pollution, enhances water efficiency, improves water quality, and uses environmentally friendly preparations that protect against pests and diseases [3]. It aims to minimize inputs and create a closed (self-sustaining) system. However, converting from conventional to organic production systems is a difficult path, as organic farming is knowledge-intensive and technically and manually demanding, especially for perennial crops such as fruit plants [4].

Organic production is organized in 191 countries, and more than 76 million ha are farmed organically by at least 3.7 million farmers [5]. Due to the consumer’s awareness of safe and healthy food, organic farming has a dramatic expansion across all crops, especially fruit, which has increased by 83.4% in the last decade. In 2021, around 302,000 ha, or 2.6% of the world’s temperate fruit area, was organically farmed, of which 156,600 ha was in Europe, the leading continent, and 112,000 ha was in China, the leading country. The most important fruits are apples (26.02%), plums (12.77%), apricots (5.08%), cherries (4.68%), pears (4.17%) and peaches (2.09%). In Norway, organic production is practiced on 216 ha, with plums being one of the most important crops [5].

Plum (*Prunus domestica*) is one of the most attractive fruit species in temperate zones as it has a high economic yield and is very popular with both producers and consumers due to its exceptional aroma, taste, and color. The fruit can be eaten fresh but is also dried or processed into brandy, preserves, compotes, mousses, pulp, candied fruit, wine, jams, and jelly products [6]. It is considered a respectable source of nutrients due to the content of minerals (K, P, Ca, Mg, Na, B, Se, Fe, Zn, Mn, Cu), vitamins (A, B, E, C, K), sugars (fructose, sucrose, glucose, sorbitol), organic acids (citric acid, malic acid), dietary fiber, phenolics (flavonoids and phenolic acids), aromatic compounds (benzaldehyde, linalool, ethyl nonanoate), and then tannins, enzymes, and proteins [7,8]. The peel has a high content of anthocyanins (cyanidins and peonidins) and carotenoids (especially β-carotene), which is influenced by the location of the tree, ripening time, shading, temperature, humidity, or soil type [9,10,11,12,13]. The plum kernel is also a rich source of polyphenols, especially protocatechuic acid, *p*-hydroxybenzoic acid, ferulic acid, and chlorogenic acid [14]. The quantity and quality of bioactive compounds in the fruit are strongly related to the genotype, soil maintenance, pesticide use, and postharvest treatments [7,15,16,17,18]. The consumption of plums is associated with a reduced incidence of degenerative diseases due to their antioxidant potential [19]. They also have anticancer, antihyperglycemic, antihypertensive, anti-allergic, and laxative properties [20]. Moreover, studies on dried plums have shown that the fruits have a positive effect on bone mass and bone microarchitecture, lower “bad” LDL cholesterol, and increase “good” HDL cholesterol [21,22,23]. In folk medicine, plums are used for their effects against fatigue, stress, and insomnia, for lowering cholesterol levels, and against asthma and anemia [24]. Dry and fresh plum extracts have shown antibacterial activity against *Staphylococcus aureus* and *Escherichia coli* [25] and antimutagenic activity against *Salmonella typhimurium* TA 98 and TA100 strains [26]. They play a role in the prevention of constipation as they contain high levels of sorbitol, which is a natural laxative [27]. Plums do not appear to cause a significant increase in blood glucose levels as they increase adiponectin levels, a hormone that plays a role in regulating blood glucose levels, and have high fiber content [28,29].

According to the literature data, plum cultivation in Norway has a long tradition, dating back to the Middle Ages [30]. Plum production is organized around lakes in the eastern part of the country and around fjords in the western part (between 58° N and 62° N), which overall represents the northern limit of commercial fruit production in the world [31]. Although the northern latitudes are under the influence of the Gulf Stream, the growing seasons is short and relatively cool [32]. As Norwegians prefer large and tasty fruits, Opal, Victoria, Edda, Mallard, Reeves, and Jubileum are the most important plum cultivars [33,34]. Organic production in Norway today accounts for 4.4% of total agricultural production, with plums accounting for 10% of organic production in the country [35].

As far as we know, this is the first comprehensive analysis of organically produced plums from Norway. The aim was therefore to determine and compare the metabolite profile of seven plum cultivars grown in two different locations.

## 2. Materials and Methods

### 2.1. Plant Material, Soil and Management

The plums used for this study were grown in western Norway at the experimental farm of NIBIO Ullensvang (60.318655, 6.652948), at growers in the Ullensvang municipalities, and on one farm in the municipality of Telemark (59.374658, 9.221676) in eastern Norway (Table 1). The locations of these orchards were typical for the main fruit production areas in Norway. For this study, only commercial varieties were selected whose acreage is representative of the total plum production in Norway. In August and September in 2022, fruit samples from seven plum cultivars were collected from different commercial organic orchards in western and eastern Norway. Out of seven cultivars, only one was an early cultivar (‘Opal’), and one was late (‘Valor’), while the rest were mid-season cultivars (‘Mallard’, ‘Reeves’, ‘Jubileum’, ‘Excalibur’ and ‘Avalon’). The number of cultivars for a season is limited. ‘Opal’ is the most important early ripening cultivar and ‘Valor’ is the most important late-ripening cultivar. Soil types and physical and chemical characteristics are already described in the literature [36,37].

All plum cultivars were grafted onto ‘St. Julien A’ rootstocks spaced 1.0–1.5 m × 3.5–4 m apart. The trees were trained as spindle trees and pruned to a maximum height of about 2.5–3 m. The organic sites were officially certified by the Norwegian inspection body Debio (Bjørkelangen, Norway) in accordance with the Norwegian regulations for the production and labeling of organic agricultural products. Organic pest protection was carried out in all orchards in accordance with the official guidelines. Weeds under the trees were removed by frequently mowing and the use of a rotator tiller. Only a few pests (rust mite (*Aculus fockeui*), European red mite (*Panonychus ulmi*), aphids (*Brachycaudus helichrysi*, *B. cardui*), and various lepidopterous larvae) are a problem in organic plum production, and the pesticide program is less demanding than in apple and pear [38]. The trees were treated with copper oxide (trade name Nordox 75WG, with 86% copper oxide as an active ingredient) against different diseases (bacterial canker, plum pouch) during bud swelling. Aphids and plum leaf mites were controlled with sulfur (trade name Thiovit Jet, with 80% sulfur as an active ingredient) and soyabean oil. The trees were fertilized with organic chicken manure (pellets), 8% N, 4% P, and 5% K as a percentage of dry matter. Drip irrigation was installed in all fields with a drip line along the tree rows with 0.5 mm drip spacing. The trees were watered regularly when a water deficit occurred due to evaporation and precipitation. On average, 2–3 mm of water was applied daily in this relatively cool climate, depending on the evaporation rate. All trees received the same amounts of fertilizer based on soil analysis. Hand thinning was carried out during June to achieve optimal harvest quantity with good fruit quality (5–7 cm distance between fruitlets). The fruits were picked when they were fully ripe. After harvesting, the fruits were placed in the dryer and dried at 40 °C for 10 days.

### 2.2. Climate Conditions

Fruit production in Norway is conducted in the south part of the country, where the climate is most favorable, as well as around lakes in the east part and in fjord areas in the west. The fjord areas in western Norway have a maritime climate with relatively cool summers and mild winters. The weather fronts usually come from the southwest, from the North Sea and the Atlantic. There are rarely problems with frost damage to the fruit trees, neither in winter nor during the blossom time. The snow-covered mountains offer protection from large amounts of rain from the west. On the other hand, the climate is the main limiting factor due to the relatively cool summers, resulting in a relatively short and cool growing season, which limits both the species and cultivars that can be grown. The climate in Ullensvang (western Norway) during 2022 was slightly warmer and drier than the 30-year average (1990–2020). According to this average, the temperature during the growing season was 12.3 °C, with 638 mm of precipitation. In 2022, the temperature was 13.5 °C, with only 380 mm of precipitation during the growing season. The climate on the eastern side (Telemark) is more continental, with warmer summers and colder winters with less rainfall. Frost can occur in winter and during the flowering period. However, the orchards are mainly located near lakes, where the water moderates the more extreme temperatures. In Midt-Telemark, the average temperature in 2022 was 15.2 °C, with 369 mm of rainfall in the growing seasons, while the long-term average (1990–2020) was 14.0 °C and 399 mm of precipitation in the growing season.

### 2.3. Reagents and Standards

Standards of sugar (glucose, fructose, sucrose, arabinose, melibiose, raffinose, maltose and panose), sugar alcohol (glycerol, sorbitol, and mannitol), organic acid (quinic, malic, citric, shikimic, galacturonic, fumaric, and maleic acid), and polyphenol (gallic acid, chlorogenic acid, catechin, caffeic acid, aesculin, rutin, p-coumaric acid, hyperoside, isorhamnetin-3-*O*-rutinoside, isorhamnetin-3-*O*-glucoside, kaempferol-3-*O*-glucoside, ellagic acid, quercetin, and isorhamnetin) were purchased from Supelco/Sigma-Aldrich (St. Louis, MO, USA). Also, we acquired Folin–Ciocalteu reagent, sodium carbonate, 50% sodium hydroxide, sodium acetate trihydrate, methanol, acetonitrile (MS grade), and formic acid (MS grade). Aqueous standard solutions were prepared with ultrapure water (0.055 µS/cm) using a MicroPure water purification system (Thermofisher TKA Germany). Syringe filters (13 mm, PTFE 0.22 μm, and 0.45 μm) were purchased from Supelco (Bellefonte, PA, USA).

### 2.4. Preparation of Sample Extracts

The polyphenol extracts of dry plum were prepared according to the previously described method [3]. Ground dry plum (approximately 0.5 g) mixed with 25 mL of methanol/water solution (70/30, *v*/*v*) containing 0.1% HCl using an ultrasonic bath for 30 min. The supernatant of each sample was collected after centrifugation at a frequency of 9000 rpm for 10 min. The extraction procedure was repeated two more times. Thereafter, all supernatants for each sample were combined and evaporated to dryness in a vacuum evaporator (IKA RV8, IKA^®^—Werke GmbH & Co. KG, Staufen, Germany). The residue after evaporation was dissolved in a solution of methanol/water (60/40, *v*/*v*) to 25 mL. Those solutions were used for the quantification of individual polyphenols and the determination of total phenolic content (TPC) and antioxidant capacity (RSA). All extractions were performed in triplicate, and extracts were filtered through 0.45 μm membrane filters (Syringe Filter, PTFE, Supelco) before analysis.

For the sugar, sugar alcohol, and organic acid analysis, 0.5 g of each sample was extracted with ultrapure water (0.055 µS/cm) using an ultrasonic bath for 30 min [3]. Next, the mixture was centrifuged at 9000 rpm for 20 min and the supernatant was used for analysis. For the quantification of glucose, fructose, and sucrose, as major sugars, prepared extracts were 100-fold diluted. All extractions were performed in triplicate, and extracts were filtered through 0.22 μm membrane filters (Syringe Filter, PTFE, Supelco) before analysis.

The procedure for preparing samples for elemental analysis is explained in detail in our previous paper [39].

### 2.5. Determination of Total Phenolic Content (TPC) and Radical-Scavenging Activity (RSA)

TPC was determined following a procedure published by Singelton and Rossi [40]. All modifications were given in detail in our previous paper [3]. RSA results were collected following the procedure described by Papetti and coworkers [41], with slight modifications given in [3]. TPC amounts were expressed as grams of gallic acid equivalent (GAE) per kg of dry weight (DW), while RSA results were expressed as mmol Trolox equivalent (TE) per kg of DW.

### 2.6. Determination of Polyphenol Profile Using UHPLC-DAD MS/MS

Amounts of individual polyphenols in investigated plum extracts were determined using a Dionex Ultimate 3000 UHPLC system (Thermo Fisher Scientific, Bremen, Germany) equipped with a diode array detector (DAD) coupled to a TSQ Quantum Access Max triple-quadrupole mass spectrometer (ThermoFisher Scientific, Bremen, Germany). The separation, determination, and quantification conditions were previously described in the literature [3]. Concentrations of the individual polyphenol compounds were expressed as mg kg^−1^ DW.

### 2.7. Analysis of Sugar, Sugar Alcohols, and Organic Acids

The quantification of sugars and sugar alcohols was conducted using a high-performance anion-exchange liquid chromatography system, Dionex ICS 3000 DP LC (Dionex, Sunnyvale, CA, USA), with pulsed amperometric detection. For the organic acids analysis, the Dionex ICS 3000 DP LC system was coupled with a conductivity detector. The performance of the ion chromatography device and the determination conditions for the quantification of sugars, sugar alcohols, and organic acids are described in our previous work [39].

### 2.8. Elemental Analysis

The quantification of major and trace elements was performed using inductively coupled plasma optical mass spectrometry (ICP-MS, Thermo Fischer Scientific, Cambridge, UK). The conditions for ICP-MS analysis were described in our previous study [39].

### 2.9. Statistical Analysis

All obtained values in the tables are shown as an arithmetic mean of three replicates. Tukey’s test was used to detect significant differences (*p* ≤ 0.05) between the mean values. Tukey’s test was performed with the statistical program MS Excel (Microsoft Office 2016 Professional). Principal component analysis (PCA) was carried out using the PLS_Tool Box software package (Version 6.2.1, Eigenvector Research, Inc., Wenatchee, WA 98801) for MATLAB (Version 7.12.0, Budapest, Hungary), as described in our previous paper, and all data were group-scaled prior to PCA [42].

## 3. Results and Discussion

### 3.1. Elemental Compositions of Investigated Dry Plum Samples

As expected, K was the most abundant element in the plum samples analyzed, with concentrations ranging from 10.9 g/kg dry weight (DW) (UO1) to 45.0 g/kg DW (UJ1) and accounting for 81.48% (TO1) up to 88.55% (UO1) of all minerals detected (Table 2). K is referred to as a “quality element” because it is one of the most important minerals required for plant growth and development [43]. This macronutrient is involved in the postharvest quality of the fruits and influences parameters such as size and color, shelf life, acidity, and nutritional value [44]. The next minerals, in order of abundance, were P, Mg, and Ca, in ranges of 0.74 g/kg (UO1)–4.9 g/kg DW (UJ1); 0.37 g/kg DW (UO1)–2.35 g/kg DW (UO3); and 0.123 g/kg DW (UO1)–1.39 DW (UJ1), respectively. The same trend of major mineral order in different *Prunus* species (*Prunus salicina* and *Prunus domestica*) was shown in previous papers [45,46,47,48]. Phosphorus (P) has been associated with fruit yield, the production of soluble solids, and secondary metabolites such as ascorbic acid and flavonoids [49]. Calcium has a central role in cell wall interactions, plant signals, and water relations, so deficiencies can lead to leaky membranes, the irregular softening of cell walls, abnormal fruit development, and even pathological and physiological fruit disorders [50]. Ozzengin and co-workers studied *Prunus domestica* var. *karaca* and *P*. *domestica* var. *uryani* grown in Turkey, and they quantified a total of eight elements, namely K, Ca, Mg, Na, Fe, Zn, Cu, and Mn, and except for Na, all amounts were lower compared to our results [51]. Among all 30 plum samples, sample UO1 stood out with the lowest values of the most quantified elements (K, P, Mg, Ca, B, Mn, Fe) and was the only sample in which Zn was below the limit of quantification (<0.5 mg/kg DW).

Samples UO3 and UJ1 stood out due to their particularly high levels of P, K, Mg, and Ca, which averaged 4692 mg/kg DW, 43,287 mg/kg DW, 2264 mg/kg DW, and 1167.2 mg/kg DW, respectively, while the average values for the same minerals in the other samples were 1883 mg/kg DW, 21,571 mg/kg DW, 854 mg/kg DW, and 589.1 mg/kg DW, respectively. Regarding microelements, among all analyzed samples, particularly higher values were found for Zn in sample UM1 (56.6 mg/kg DW), Fe in sample UM6 (55.4 mg/kg DW), B in TM1 (58.1 mg/kg DW), and Mn and Cu in UO3 (22.4 mg/kg DW and 11 mg/kg DW, respectively). Iron is associated with biomass, chlorophyll, and photosynthesis in most cases, but may lead to lower fruit production and fruit quality in stone fruits due to the lower ratio of total sugar to total organic acid and the lower anthocyanin accumulation [52]. The metals Al, Ni, Cr, Sr, and Ba were not detected in all samples; Al was missing in 9 samples, Ni was missing in 13 samples (including all Telemark samples), and Cr was missing in 14 samples. The highest Al content was found in UM3 (73 mg/kg DW), the highest Ni content was in UM1 (5.1 mg/kg DW), the highest Sr content was in TV1 (20 mg/kg DW), and the highest Ba content was in UO6 (9.5 mg/kg DW). A significantly high Cr content (7.7 mg/kg) was found in sample UR1 compared to the other samples, where the average Cr content was 1.03 mg/kg DW (Table 2).

In terms of location, plums from Ullensvang had higher levels of K, Mg, Ca, Na, and Zn, while fruit from Telemark had higher levels of B, Fe, and Cu. The soils originating from different bedrocks are characterized by different geochemistry, which could be the reason for the different mineral content of the plum fruits from two distant locations. Both Ba and Sr are widespread elements that occur in significant amounts in Earth’s crust and are closely related to the nature of the given bedrock [53]. They have the potential to influence plant growth directly or indirectly by competing with and or replacing essential elements such as calcium and magnesium [54]. Their uptake and precipitation differ between soil types, plant species, tissue age, tissues within an individual plant, and environmental factors [55]. In contrast to calcium and magnesium, strontium and barium are not essential for plant growth and can even be toxic to plants [56]. At a high level of S in leaves (S is used as a fungicide in organic production, and mostly for foliar applications), sulfate ions react with Sr and Ba, sulfate crystals are formed, and homeostasis in plant cells is maintained [55,57]. Finley and Hudgins and co-authors believe that these crystals, depending on their shape, size, placement, and abundance, can prevent plant feeding by large animals and insects [58,59]. Plum fruits from Ullensvang had ~2-fold higher Ba content, while plums from Telemark had ~1.6-fold higher Sr content; this information can serve as a biomarker for the location where the plums were grown. This all implies that different locations had different soil types.

Regardless of the location, the Opal cultivar had the highest Na content; Mallard had the highest K and Zn content; Excalibur had the highest Mn, Cu, and Al content; Avalon had the highest P, B, and Fe content; and finally, Avalon had the highest Mg and Ca content. In general, the elemental composition of plum fruit depends on environmental conditions such as soil type and texture, soil nutrient content and its ratio, rainfall, field water capacity, air temperature, horticultural practices, and other aspects [60].

### 3.2. Determination of Sugars and Sugar Alcohols

Sugars are primary metabolic products obtained through the process of photosynthesis; they provide energy and are used as carbon building blocks for biochemical processes. The sugar composition is important for the sweetness of the fruit and consumer acceptance [61]. The ripening process, age of the plant, soil characteristics, microclimatic conditions, agrotechnical measurements, and cultivar influence the quantitative variations of sugars in the fruit [62,63].

In all plum samples analyzed, nine sugars and three sugar alcohols (Table 3) were determined. The dominant sugars in plum samples were glucose, fructose, and sucrose, which had already been detected in many other plum cultivars [64,65]. Considering sugar alcohols, sorbitol was the most common sugar in the plums examined. Sorbitol and glucose, which are formed from the products of photosynthesis in the leaves, are the translocation sugars that pass through the phloem into the fruit tissue, where they are converted into fructose, malic acid, or starch, depending on the stage of development [66]. Sorbitol is preferentially converted into fructose, while glucose is preferentially incorporated into starch [67,68].

The glucose content ranged from 19.18 g/100 g DW (TJ1) to 26.88 g/100 g DW (UO3); fructose ranged from 9.34 g/100 g DW (UJ3) to 20.43 g/100 g DW (TO1); sucrose ranged from 0.12 g/100 g DW (UE1) to 2.73 g/100 g DW (UM6); and sorbitol content ranged from 8.45 g/100 g DW (TJ1) to 17.20 g/100 g DW (UO3), which corresponds to the results of [8,19,69]. Glucose accounted for up to 47.04% (UJ1), fructose accounted for up to 36.84% (TO1), sorbitol accounted for up to 29.39% (UM1), and sucrose accounted for up to 5.75% (TR1) of all quantified sugars. It was expected that the sucrose content would be low due to its hydrolysis of glucose and fructose. These four sugars together accounted for between 89.42% (TJ1) and 97.00% (UO4). Hartmann divided the plum cultivars into three groups according to their sorbitol content in dry weight: plums with a low sorbitol content (0.2 to 5.6%); plums with a medium sorbitol content (3.3 to 6%); and plums with a high sorbitol content (6.6 to 35.1%), with all the plum cultivars examined belonging to the last group [70]. The sum of the minor sugars (arabinose, melibiose, raffinose, maltose, panose, glycerol, and mannitol) ranged from 1.43 g/100 g DW (UJ1) to 5.15 g/100 g DW (UO2).

The total amount of quantified sugars ranged from 47.39 g/100 g DW (UJ1) to 61.43 g/100 g DW (UO1). In general, the Opal and Mallard cultivars had a much higher sugar content compared to Jubileum, Excalibur, Avalon, and Valor. According to the results, plum fruits with higher levels of glucose and sorbitol content were produced in the Ullensvang area than in the Telemark area, while fructose content was higher in the Telemark area. Regardless of the location, Excalibur fruit had the highest fructose content (15.65 g/100 g DW) but the lowest sorbitol content (10.26 g/100 g DW), and a sucrose content of 0.12 g/100 g DW (although we only analyzed one sample from Telemark). Opal had the highest sorbitol content (15.17 g/100 g DW), while Mallard had the highest glucose and sucrose content (24.59 g/100 g DW and 2.12 g/100 g DW, respectively).

### 3.3. Determination of Organic Acids

During fruit development, the organic acid content is generally in inverse proportion to the sugar content. When maturing, sugars accumulate, mainly due to sugar import or from starch degradation, while organic acids that have accumulated in young fruits are greatly reduced [61]. In addition to sugars, the diversity and concentrations of organic acids also contribute significantly to the organoleptic properties of plums, such as flavor and quality [71]. The proportions of the individual acids are also important as citric acid masks the perception of sucrose and fructose, while malic acid seems to improve the perception of sucrose [72].

The total acid content of the plum fruits tested ranged from 7.72 g/kg dw (TO1) to 21.15 g/kg dw (UJ1). In the plum samples studied, quinic acid was the most abundant organic acid (accounted for, on average, 57.1%), followed by malic (17.91%), and citric acid (11.1%) (Table 4). This does not correspond with the studies of Stacewicz-Sapuntzakis and co-workers [19] and Lin and co-authors [73], who claimed that malic acid was the dominant one. Quinic acid was the most abundant organic acid in the samples, mainly because it is involved in plant biosynthesis processes, and its content ranged from 2.72 (TR2) to 13.70 g/kg dw (UJ1). It serves as a precursor for the biosynthesis of polyphenols, such as chlorogenic acids and flavonoids, in plants [74]. In this study, its contribution to the total acid content ranged from 32.75% (UO2) to 68.40% (UO4). On the other hand, the proportion of malic acid was between 5.96% (UM1) and 30.06% (TA1). The results obtained in this study are in accordance with the results from our previous work [6]. Samples from the Ullensvang area showed higher contents of quinic acid, citric acid, and maleic acid than samples from the Telemark area, which contained higher contents of shikimic acid, galacturonic acid, fumaric acid, and malic acid.

The average organic acid content in plums from the Telemark area was 9.95 g/kg dw, while the average organic acid content in plum samples from the Ullensvang area was 11.40 g/kg. From the results, it can be concluded that Norwegian plums are generally more acidic, which is consistent with the literature [6,33]. Regardless of the location, the cultivar Jubileum had the highest acidity (13.56 g/kg dw), and Avalon had the lowest (8.17 g/kg dw). The plum cultivar Opal, on average, had the highest level of citric acid (1.94 g/kg dw), and the Mallard cultivar had the highest level of shikimic acid (1.26 g/kg dw), galacturonic acid (2.57 g/kg dw), and maleic acid (0.45 g/kg dw). Jubileum had the highest average content of quinic acid (8.32 g/kg dw) while Valor had the highest average content of malic acid (3.31 g/kg dw). Also, according to the results of the study by Stacewicz-Sapuntzakis and co-authors, dehydration does not change the acidity of prunes, as their pH remains stable at 3.6 even during drying [19].

According to Li and co-authors, the ratio of malic to citric acid could be an important index for the differentiation and authentication of fruits and juices [75]. In this study, this ratio was between 0.41 (UO4) and 4.15 (TV1), which is comparable to peach and pear fruits and their juices according to previous authors.

### 3.4. Amounts of Individual Polyphenols, TPC, and RSA

A total of fifteen polyphenols were quantified in the dried plum samples analyzed (Table 5). Eight polyphenols (chlorogenic acid, caffeic acid, rutin, hyperoside, isorhamnetin-3-*O*-glucoside, kaempferol-3-*O*-glucoside, quercetin, and isorhamnetin) were quantified in all examined samples. Only in one sample, UJ1, all 15 polyphenols were quantified. Chlorogenic acid was the most abundant polyphenol in all samples analyzed (141.9 mg kg^−1^ on average), with the exception of sample UM4, in which gallic acid (10.67 mg kg^−1^) was the most abundant polyphenol, and sample UO2, in which kaempferol-3-*O*-glucosides (286.21 mg kg^−1^) was the most abundant polyphenol. The highest amount of chlorogenic acid (503.90 mg kg^−1^) was determined in sample TV1. Besides chlorogenic acid, the polyphenols with the highest average contents were kaempferol-3-*O*-glucoside (average 26.47 mg kg^−1^) and rutin (average 25.90 mg kg^−1^). Similar results for chlorogenic acid and rutin have already been found in the literature [76,77,78,79]. Chlorogenic acid, together with kaempferol-3-*O*-glucoside and rutin, accounted for 31.22% (UM4) to 86.58% (UO1) of total quantified polyphenols. According to Donovan and co-workers, chlorogenic acid represented 24% of all phenolic compounds in the dry mesocarp of plum fruit, while rutin accounted for 2% of all phenolics [80].

If the sum of the quantified individual polyphenols is compared, three samples stand out: UO2, UJ1, and TV1, in which the sum of the individual polyphenols amounted to 880.24 mg kg^−1^, 657.38 mg kg^−1^, and 763.29 mg kg^−1^, respectively. The highest contents of gallic acid, catechin, hyperoside, isorhamnetin-3-*O*-glucoside, kaempferol-3-*O*-glucoside, quercetin-3-*O*-rhamnoside, and ellagic acid were determined in sample UO2. Sample UJ1 contained higher amounts of caffeic acid, coumaric acid, isorhamnetin-3-*O*-glucoside, quercetin, and isorhamnetin compared to the other samples. The highest amounts of chlorogenic acid and rutin were quantified in the Valor cultivar grown in Telemark (TV1). In contrast, the lowest sum of quantified polyphenols (45.32 mg kg^−1^) was determined in sample UM4. Plum fruit from Telemark showed higher polyphenol content compared to that from Ullensvang. Regardless of location, Valor had the highest polyphenol content, while Excalibur had the lowest. A similar profile of polyphenols in plums falls within a wide range of values, which is already described in the literature [81,82,83]. The TPC value ranged from 4.43 g GAE/kg (UM4) to 16.24 g GAE/kg (UJ1), with an average value of 8.66 g GAE/kg. These results are higher than those obtained by Sohair and co-workers for air-dried plums [84]. The RSA values were between 35.42 mmol TE/kg (UM6) and 262.91 mmol TE/kg (UM1), and the average activity was 111.82 mmol TE/kg. Plums from Ullensvang had higher TPC and RSA values, while Valor had the highest TPC value and Jubileum had the highest RSA value. The results obtained are in fairly close agreement with the Norwegian plums analyzed by Fotirić Akšić and co-authors [6], together with those investigated in other studies [73,85].

### 3.5. Principal Component Analysis (PCA)

In order to determine whether the biological/geographical origin had an influence on the differences in the chemical composition of the plum samples examined, PCA was first applied to all the results obtained in this study. However, as the model obtained was not satisfactory and the presence of clusters could not be demonstrated, PCA was applied separately to four different data sets (phenolic amounts, TPC, and RSA; elemental compositions; organic acid contents; sugars and sugar alcohols) similar to our previous research [39].

PCA of polyphenols, TPC, and RSA resulted in a model where the first two PCs described 64.5% of the total variability. The PCA score plot (Figure S1A) showed that there was no clustering among the samples, almost all samples were in the middle, except for two samples that were outside Hotelling’s T2 ellipse with 95% probability, i.e., they were outliers. Sample UJ1 (Jubileum from Ullensvang, orchard no. 1) was separated from other samples based on the highest content of caffeic acid, coumaric acid, quercetin, and isorhamnetin (Figure S1B). On the other hand, the high content of gallic acid, catechin, hyperoside, isorhamnetin-3-O-glucoside, kaempherol-3-O-glucoside, quercetin-3-O-rhamnoside, and ellagic acid was mainly responsible for separating sample UO2 (Opal from Ullensvang, orchard no. 2) from the other samples.

PCA performed for major and minor elements resulted in a three-component model, in which the first three components described 61.9% of the variability (PC1, PC2, and PC3 accounted for 36.2%, 14.8%, and 10.9%, respectively). Although the resulting model (Figure S1C,D) did not show clustering based on biological/geographical origin, some samples stood out from the rest, and three outliers were identified. Samples UR1 (Reeves from Ullensvang, orchard no. 1) and UM1 (Mallard from Ullensvang, orchard no. 1) differed from the other plum samples due to their higher content of Ni. The most significant influence for the differentiation of sample UO3 (Opal from Ullensvang, orchard no. 3) from the other plum samples was the higher content of Mn, Mg, K, and P compared to most other samples. The PC score diagram (Figure S1C) also shows that the Opal samples from Ullensvang (UO1, UO2, UO4, UO5, and UO6), together with Opal sample from Telemark (TO1), form a cluster that was separated from other samples based on the higher Na content.

PCA applied to sugar and sugar alcohol content resulted in a model in which the first three components described only 59.8% of the variability (PC1, PC2, and PC3 account for 29.4, 16.8, and 13.6% of the total variability). The PCA score plot (Figure S1E) shows that there is no clear separation based on cultivar or geographical origin. However, some samples were separated from the others (Figure S1E). Several samples from Telemark (TO1, TM1, and TJ1), together with one sample from Ullensvang (UR4) were separated due to their higher mannitol content compared to the other plum samples (Figure S1F).

**Figure 1:**
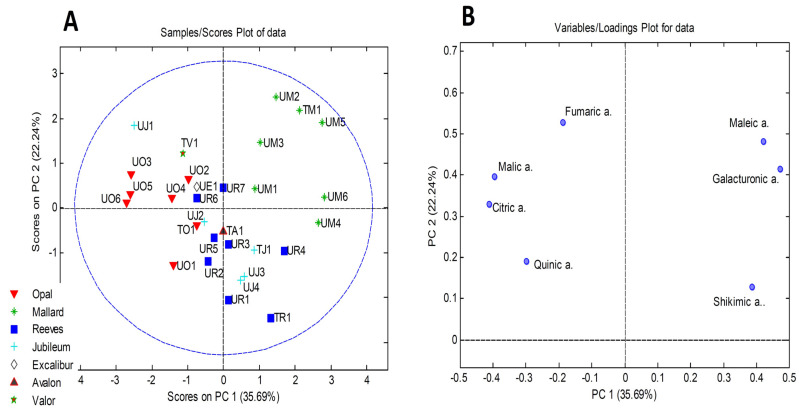
PCA performed on organic acid content quantified in plum samples: (**A**) PCA score plot and (**B**) PCA loading plot (“a.”–stands for acid).

The clearest grouping was observed when PCA was applied to the organic acid content. The PCA score plot and loading plot are presented in Figure 1A,B. In the PCA model obtained, the first three principal components explained 75.3% of the variability. The Mallard samples from both sites (UM1, UM2, UM3, UM4, UM5, UM6, and TM1) were separated based on a higher content of maleic and galacturonic acid. A higher content of citric acid quantified in Opal samples from Ullensvang was responsible for separating these samples from other plum samples.

Although PCA showed no clear influence of geographical/biological origin on the chemical composition of the plum samples, some groupings were observed, and the separation of individual samples based on chemical composition became clearer.

## 4. Conclusions

Due to the increasing interest in functional foods produced on the basis of natural bioactive ingredients, the investigation of raw materials and the selection of suitable species/cultivars/locations with the highest possible content of such metabolites are desirable. To our knowledge, this is the first study to provide a comprehensive and comparable analysis of organically grown plums in Norway. The content of minerals, sugars, organic acids, TPC, RSA, and polyphenols varied widely, which can be explained by the fact that seven different plum cultivars were used in two widely separated locations, so differences in the biosynthesis of the compounds were to be expected. In addition, the results were used to determine the authenticity of the locations and cultivars.

The results of this study summarize that among the minerals, P, K, Ca, and Mg are the most abundant, while Ba and Sr drew a clear line between the locations. All cultivars had the highest level of glucose, followed by fructose and sorbitol. Quinic and malic acid were predominant organic acids, together accounting for up to 80% of all quantified organic acids. Chlorogenic acid was predominant and shared up to 78% of all quantified polyphenols. Based on the calculated parameters, plums from Ullensvang had a higher content of minerals, sugars, organic acids, TPC, and RSA, while plums from Telemark had a higher content of quantified polyphenols. Of the cultivars tested, ‘Mallard’ stored the highest levels of minerals and RSA, ‘Opal’ had the sweetest fruits, ‘Jubileum’ had the most acidic fruits, ‘Excalibur’ had the highest TPC content, and ‘Valor’ was characterized by the highest content of quantified polyphenols, especially chlorogenic acid.

Due to the high content of stored metabolites, the data obtained represent the plum as a value-added component for functional food. In addition, all this information is fundamental for the creation of a database that could make it possible to improve the utilization of plum genetic resources in breeding programs. All data can also be of great interest for locations with similar climate and soil conditions worldwide.

## Figures and Tables

**Table 1:** Geographical, biological origin, and harvest date of plum samples.

Location	Cultivar	Orchard	Sample ID	Harvest Time
Ullensvang	Opal	#1	UO1	early
		#2	UO2	early
		#3	UO3	early
		#4	UO4	early
		#5	UO5	early
		#6	UO6	early
	Mallard	#1	UM1	mid
		#2	UM2	mid
		#3	UM3	mid
		#4	UM4	mid
		#5	UM5	mid
		#6	UM6	mid
	Reeves	#1	UR1	mid
		#2	UR2	mid
		#3	UR3	mid
		#4	UR4	mid
		#5	UR5	mid
		#6	UR6	mid
		#7	UR7	mid
	Jubileum	#1	UJ1	mid
		#2	UJ2	mid
		#3	UJ3	mid
		#4	UJ4	mid
	Excalibur	#1	UE1	mid
Telemark	Opal	#1	TO1	mid
	Mallard	#1	TM1	mid
	Reeves	#1	TR1	mid
	Jubileum	#1	TJ1	mid
	Avalon	#1	TA1	mid
	Valor	#1	TV1	late

**Table 2:** Elemental composition * of investigated dry plum samples (mg kg^−1^ DW).

Sample ID	P	K	Mg	Ca	B	Na	Mn	Fe	Cu	Zn	Al	Ni	Cr	Sr	Ba
UO1	742.4 ^w^**	10,921.9 ^w^	371.2 ^v^	128.9 ^s^	7.7 ^u^	147.9 ^d^	2.3 ^t^	2.9 ^v^	4.2 ^o^	<0.5	<0.5	<0.5	<0.5	0.6 ^v^	1.0 ^q^
UO2	1051.0 ^v^	13,805.1 ^u^	631.9 ^t^	407.2 ^p^	21.5 ^m^	244.3 ^a^	4.5 ^s^	4.5 ^u^	3.6 ^q^	3.5 s	<0.5	0.6 ^i^	<0.5	3.8 ^p^	3.9 ^g^
UO3	4456.9 ^b^	41,572.1 ^b^	2354.6 ^a^	946.1 ^c^	16.5 ^p^	19.6 ^o^	22.4 ^a^	37.2 ^c^	11.0 ^a^	25.7 ^b^	2.7 ^i^	1.0 ^f^	1.1 ^d^	10.7 ^b^	3.2 ^i^
UO4	1220.6 ^u^	13,916.7 ^u^	581.7 ^u^	428.5 ^o^	24.8 ^j^	52.5 ^g^	4.3 ^s^	5.6 ^t^	5.9 ^j^	2.9 ^t^	<0.5	1.3 ^c^	<0.5	1.2 ^u^	3.2 ^i^
UO5	2255.1 ^g^	23,921.7 ^i^	1034.8 ^f^	795.8 ^f^	35.0 ^f^	235.7 ^b^	8.5 ^l^	11.9 ^r^	6.2 ^i^	8.3 ^m^	<0.5	0.6 ^i^	0.6 ^h^	4.6 ^o^	1.3 ^o^
UO6	1968.4 ^k^	21,750.6 ^n^	1026.4 ^g^	844.0 ^e^	40.3 ^c^	84.6 ^f^	8.0 ^m^	6.8 ^s^	5.0 ^l^	5.5 ^p^	<0.5	1.0 ^f^	<0.5	7.4 ^gh^	9.5 ^a^
UM1	1677.6 ^r^	19,422.4 ^s^	832.3 ^m^	529.5 ^k^	33.6 ^g^	46.5 ^hi^	6.1 ^p^	16.4 ^n^	3.9 ^p^	56.6 ^a^	14.8 ^d^	5.1 ^a^	0.8 ^f^	7.1 ^i^	1.3 ^o^
UM2	2946.7 ^d^	36,735.6 ^c^	1505.9 ^c^	973.3 ^b^	24.8 ^j^	19.7 ^o^	19.9 ^b^	21.0 ^j^	9.6 ^c^	9.7 ^k^	<0.5	0.9 ^g^	0.8 ^f^	10.0 ^c^	8.7 ^b^
UM3	2127.7 ^i^	25,574.7 ^g^	921.1 ^i^	565.3 ^i^	23.4 ^k^	42.1 ^k^	11.0 ^f^	13.9 ^p^	7.2 ^f^	17.6 ^c^	73.0 ^a^	1.1 ^e^	<0.5	7.5 ^g^	8.6 ^b^
UM4	1608.1 ^s^	22,231.8 ^m^	884.9 ^j^	468.3 ^n^	35.7 ^e^	31.7 ^m^	10.5 ^gh^	15.6 ^o^	4.9 ^lm^	9.0 ^l^	6.1 ^f^	1.1 ^e^	0.6 ^h^	7.3 ^h^	5.5 ^d^
UM5	1873.6 ^l^	27,830.0 ^e^	1033.2 ^f^	606.5 ^h^	15.0 ^r^	35.6 ^l^	9.3 ^k^	15.6 ^o^	4.6 ^n^	8.0 ^n^	2.6 ^i^	<0.5	<0.5	4.7 ^n^	1.9 ^lm^
UM6	2022.8 ^j^	23,834.5 ^j^	964.4 ^h^	524.1 ^k^	15.8 ^q^	13.5 ^r^	14.8 ^d^	55.4 ^a^	5.6 ^k^	11.8 ^g^	48.3 ^b^	0.9 ^g^	0.8 ^f^	9.0 ^e^	7.9 ^c^
UR1	1792.5 ^o^	20,587.9 ^q^	681.0 ^r^	428.7 ^o^	10.6	47.0 ^h^	7.2 ^n^	44.4 ^b^	6.0 ^j^	14.6 ^d^	3.1 ^h^	3.5 ^b^	7.7 ^a^	2.3 ^t^	2.2 ^k^
UR2	1882.8 ^l^	21,996.7 ^n^	720.1 ^q^	406.9 ^p^	23.3 ^k^	33.3 ^m^	8.6 ^l^	24.3 ^g^	4.2 ^o^	13.0 ^e^	1.9 ^l^	1.1 ^e^	1.6 ^c^	3.1 ^r^	1.8 ^m^
UR3	1720.1 ^p^	20,224.4 ^q^	784.8 ^o^	538.5 ^j^	24.4 ^j^	18.2 ^p^	7.9 ^m^	16.0 ^n^	5.0 ^l^	10.4 ^j^	<0.5	<0.5	0.6 ^h^	4.9 ^m^	2.0 ^l^
UR4	2039.4 ^j^	24,135.2 ^h^	814.9 ^n^	490.5 ^m^	29.1 ^i^	8.5 ^u^	10.3 ^h^	23.7 ^h^	8.8 ^d^	9.0 ^l^	3.2 ^h^	0.9 ^g^	<0.5	4.5 ^o^	1.2 ^p^
UR5	1700.4 ^q^	21,167.6 ^o^	729.4 ^q^	379.0 ^q^	13.5 ^s^	5.9 ^w^	9.7 ^j^	2.03 ^w^	7.0 ^g^	18.0 ^c^	0.7 ^o^	<0.5	0.7 ^g^	2.8 ^s^	0.6 ^t^
UR6	2153.4 ^h^	25,838.8 ^f^	863.2 ^k^	527.2 ^k^	33.8 ^g^	46.3 ^i^	10.3 ^h^	17.8 ^l^	7.5 ^e^	11.4 ^h^	0.7 ^o^	1.2 ^d^	<0.5	6.3 ^k^	7.7 ^c^
UR7	2125.5 ^h^	20,437.8 ^q^	840.2 ^l^	495.7 ^l^	22.7 ^l^	43.7 ^j^	10.7 ^g^	22.3 ^i^	6.8 ^h^	12.8 ^e^	5.1 ^g^	<0.5	<0.5	4.7 ^n^	0.9 ^r^
UJ1	4927.1 ^a^	45,002.4 ^a^	2173.1 ^b^	1388.3 ^a^	17.1 ^o^	19.7 ^o^	12.0 ^d^	29.3 ^c^	4.0 ^p^	10.3 ^j^	1.2 ^n^	<0.5	1.0 ^e^	7.9 ^f^	3.4 ^h^
UJ2	1939.3 ^l^	17,573.0 ^t^	830.0 ^m^	791.5 ^f^	17.9 ^n^	14.6 ^q^	6.6 ^o^	22.5 ^i^	4.8 ^m^	9.5 ^k^	1.5 ^m^	0.8 ^h^	1.7 ^bc^	3.6 ^q^	2.3 ^k^
UJ3	1847.1 ^m^	19,918.9 ^r^	776.7 ^o^	556.8 ^i^	8.4 ^t^	6.6 ^v^	7.1 ^n^	16.9 ^m^	5.9 ^j^	5.7 ^o^	2.2 ^jk^	<0.5	0.6 ^h^	3.0 ^r^	4.0 ^g^
UJ4	1301.1 ^t^	17,471.6 ^t^	665.4 ^s^	496.2 ^l^	23.1 ^k^	9.3 ^t^	5.9 ^q^	11.9 ^r^	4.5 ^n^	5.2 ^q^	2.1 ^k^	0.5 ^j^	<0.5	5.7 ^l^	2.7 ^j^
UE1	2371.4 ^f^	23,142.0 ^k^	876.4 ^k^	759.4 ^g^	15.7 ^q^	92.6 ^e^	21.3 ^a^	17.3 ^lm^	9.4 ^c^	14.2 ^d^	21.3 ^c^	0.6 ^i^	<0.5	4.3 ^p^	4.5 ^f^
TO1	1454.1	12,386.6 ^v^	587.0 ^u^	471.1 ^n^	46.5 ^b^	212.2 ^c^	5.7 ^qr^	20.0 ^k^	6.2 ^i^	4.2 ^r^	<0.5	<0.5	<0.5	4.9 ^m^	0.8 ^s^
TM1	2970.3 ^c^	32,324.8 ^d^	1332.3 ^d^	707.4 ^h^	58.1 ^a^	11.0 ^s^	17.0 ^c^	25.6 ^e^	10.4 ^b^	11.1 ^i^	<0.5	<0.5	1.8 ^b^	9.5 ^d^	1.5 ^n^
TR1	1822.2 ^n^	22,727.5 ^l^	822.5 ^n^	322.2 ^r^	22.5 ^l^	4.9 ^x^	10.1 ^i^	26.3 ^d^	5.6 ^k^	12.3 ^f^	1.9 ^l^	<0.5	1.1 ^d^	6.8 ^j^	1.4 ^no^
TJ1	1850.1 ^m^	19,969.2 ^r^	742.8 ^p^	382.8 ^q^	33.2 ^h^	25.2 ^n^	5.6 ^r^	12.8 ^q^	3.9 ^p^	8.1 ^mn^	2.3 ^j^	<0.5	<0.5	6.3 ^k^	0.8 ^s^
TA1	2397.3 ^e^	23,133.7 ^k^	886.1 ^j^	408.3 ^p^	37.5 ^d^	9.3 ^t^	11.7 ^e^	24.8 ^f^	7.2 ^f^	9.5 ^k^	14.8 ^d^	<0.5	1.7 ^bc^	4.5 ^o^	1.1 ^q^
TV1	1876.1 ^l^	21,014.4 ^p^	1168.3 ^e^	905.0 ^d^	16.4	42.6 ^k^	10.0 ^i^	17.6 ^l^	6.9 ^gh^	13.3 ^e^	11.2 ^e^	<0.5	<0.5	20.0 ^a^	4.8 ^e^

* Li, Co, As, Se, Sn, Cd, Sb, Hg, and Pb were below the LOQ (<0.5 mg kg^−1^); ** Different letters within the same column indicate statistically significant differences at *p* < 0.05 by Tukey’s test.

**Table 3:** Contents of sugars and sugar alcohols (g/100 g DW).

Sample ID	Glucose	Fructose	Sucrose	Glycerol	Sorbitol	Mannitol	Arabinose	Melibiose	Raffinose	Maltose	Panose
UO1	23.06 ^i^*	16.99 ^d^	2.04 ^e^	0.32 ^i^	15.91 ^e^	0.08 ^r^	0.06 ^j^	0.23 ^m^	1.11 ^f^	0.57 ^e^	1.06 ^de^
UO2	22.09 ^l^	12.96 ^l^	1.28 ^m^	0.52 ^e^	16.63 ^b^	0.42 ^j^	0.14 ^d^	0.29 ^jk^	1.15 ^c^	1.53 ^a^	1.10 ^b^
UO3	26.88 ^b^	9.43 ^s^	1.43 ^k^	0.73 ^a^	17.20 ^a^	0.34 ^m^	0.72 ^a^	0.25 ^l^	1.15 ^c^	0.21 ^o^	0.33 ^r^
UO4	25.95 ^cd^	17.23 ^bc^	2.06 ^e^	0.44 ^f^	14.32 ^i^	0.36 ^l^	0.07 ^i^	0.02 ^u^	0.11 ^q^	0.51 ^f^	0.33 ^r^
UO5	22.61 ^j^	14.55 ^j^	1.12 ^o^	0.20 ^kl^	16.60 ^b^	0.65 ^g^	0.18 ^d^	0.22 ^mn^	0.22 ^n^	1.04 ^c^	0.30 ^s^
UO6	26.20 ^c^	12.51 ^mn^	1.76 ^i^	0.22 ^j^	16.20 ^d^	0.21 ^o^	0.24 ^b^	0.12 ^p^	0.13 ^p^	1.22 ^b^	0.26 ^t^
UM1	24.03 ^g^	12.31 ^n^	1.59 ^j^	0.74 ^a^	16.49 ^c^	0.45 ^i^	0.08 ^h^	0.02 ^u^	0.05 ^r^	0.21 ^o^	0.14 ^v^
UM2	28.59 ^a^	11.80 ^o^	2.17 ^d^	0.58 ^c^	15.74 ^ef^	0.59 ^h^	0.08 ^h^	0.05 ^t^	0.34 ^l^	0.22 ^n^	0.30 ^s^
UM3	26.32 ^bc^	11.36 ^q^	1.87 ^g^	0.54 ^d^	15.50 ^fg^	0.04	0.04 ^l^	0.43 ^f^	0.57 ^k^	1.20 ^b^	0.44 ^o^
UM4	25.30 ^e^	11.67 ^p^	1.76 ^i^	0.32 ^i^	14.95 ^h^	0.39 ^k^	0.03 ^m^	0.21 ^n^	0.97 ^j^	0.25 ^m^	0.95 ^g^
UM5	22.05 ^l^	17.41 ^b^	2.16 ^d^	0.21 ^jk^	14.84 ^h^	0.97 ^d^	0.12 ^e^	0.36 ^h^	1.09 ^g^	0.36 ^i^	1.07 ^cd^
UM6	25.62 ^de^	17.09 ^cd^	2.73 ^b^	0.12 ^p^	15.34 ^g^	0.46 ^i^	0.04 ^l^	0.28 ^k^	1.14 ^d^	1.02 ^c^	0.61 ^k^
UR1	22.73 ^j^	11.77 ^op^	1.14 ^o^	0.43 ^f^	14.48 ^i^	0.13 ^p^	0.04 ^l^	0.23 ^m^	1.07 ^h^	0.34 ^j^	0.90 ^h^
UR2	24.65 ^f^	16.33 ^e^	0.67 ^s^	0.05 ^r^	11.20 ^l^	0.40 ^k^	0.02 ^n^	0.36 ^h^	1.14 ^d^	0.27 ^l^	0.60 ^k^
UR3	23.23 ^i^	15.72 ^g^	1.95 ^f^	0.17 ^n^	10.60 ^mn^	0.60 ^h^	0.01 ^o^	0.45 ^e^	1.04 ^i^	0.41 ^h^	1.01 ^f^
UR4	22.68 ^j^	15.96 ^f^	1.13 ^o^	0.53 ^de^	9.48 ^p^	1.61 ^a^	0.11 ^f^	0.10 ^q^	1.14 ^d^	0.32 ^k^	0.95 ^g^
UR5	24.02 ^g^	15.39 ^h^	1.21 ^n^	0.15 ^o^	12.74 ^j^	0.06 ^s^	0.05 ^k^	0.99 ^a^	1.14 ^d^	0.64 ^d^	0.53 ^l^
UR6	20.72	14.97 ^i^	1.56 ^j^	0.18 ^mn^	11.70 ^k^	0.05 ^t^	0.02 ^n^	0.52 ^c^	1.11 ^f^	0.70 ^c^	0.23 ^u^
UR7	21.74 ^m^	15.94 ^f^	1.12 ^o^	0.34 ^h^	9.12 ^r^	0.43 ^j^	0.03 ^m^	0.47 ^d^	1.44 ^a^	0.22 ^n^	0.31 ^s^
UJ1	22.29 ^k^	11.31 ^q^	1.06 ^p^	0.31 ^i^	11.30 ^l^	0.40 ^k^	0.22 ^c^	0.07 ^r^	0.15 ^o^	0.22 ^n^	0.06 ^w^
UJ2	23.64 ^h^	12.69 ^m^	1.81 ^h^	0.02 ^s^	11.80 ^k^	0.12 ^p^	0.05 ^k^	0.31 ^i^	1.10 ^fg^	0.19 ^q^	0.78 ^j^
UJ3	20.41 ^o^	9.34 ^s^	1.34 ^l^	0.09 ^q^	10.07 ^o^	0.69 ^f^	0.06 ^j^	0.30 ^ij^	1.10 ^fg^	0.44 ^g^	1.12 ^a^
UJ4	22.81 ^j^	10.44 ^r^	0.47 ^t^	0.16 ^o^	12.50 ^j^	0.73 ^e^	0.09 ^g^	0.24 ^l^	1.11 ^f^	0.21 ^o^	0.85 ^i^
UE1	19.77 ^q^	15.65 ^g^	0.12 ^u^	0.19 ^lm^	10.26 ^no^	0.10 ^q^	0.03 ^m^	0.42 ^f^	1.09 ^g^	0.36 ^i^	0.49 ^m^
TO1	20.72 ^n^	20.43 ^a^	0.97 ^q^	0.41 ^g^	9.35 ^q^	0.93 ^d^	0.09 ^g^	0.22 ^mn^	1.14 ^d^	0.14 ^r^	1.05 ^e^
TM1	20.24 ^o^	16.96 ^d^	2.56 ^c^	0.67 ^b^	10.05 ^o^	1.03 ^c^	0.09 ^g^	0.38 ^g^	1.09 ^g^	0.05 ^s^	0.36 ^q^
TR1	22.71 ^j^	13.04 ^l^	2.81 ^a^	0.09 ^q^	8.80 ^s^	0.46 ^i^	0.03 ^m^	0.06 ^s^	0.30 ^m^	0.20 ^p^	0.38 ^p^
TJ1	19.18 ^r^	16.25 ^e^	1.36 ^l^	0.60 ^c^	8.45 ^t^	1.31 ^b^	0.04 ^l^	0.68 ^b^	1.13 ^e^	0.51 ^f^	1.08 ^c^
TA1	19.73 ^q^	15.30 ^h^	1.75 ^i^	0.34 ^h^	10.46 ^n^	0.32 ^n^	0.04 ^l^	0.19 ^o^	1.17 ^b^	0.22 ^n^	0.46 ^n^
TV1	20.03 ^p^	14.29 ^k^	0.73 ^r^	0.22 ^j^	10.88 ^m^	0.37 ^l^	0.02 ^n^	0.49 ^d^	1.16 ^b^	0.22 ^n^	0.52 ^l^

* Different letters within the same column indicate statistically significant differences at *p* < 0.05 by Tukey’s test.

**Table 4:** Amounts of organic acid (g kg^−1^).

Sample ID	Quinic Acid	Malic Acid	Citric Acid	Shikimic Acid	Galacturonic Acid	Fumaric Acid	Maleic Acid
UO1	3.86 ^p^*	2.01 ^k^	1.79 ^e^	0.18 ^r^	-	0.15 ^j^	0.03 ^q^
UO2	3.49 ^s^	2.96 ^c^	1.98 ^d^	1.73 ^c^	0.19 ^n^	0.25 ^de^	0.07 ^n^
UO3	6.87 ^g^	3.23 ^b^	2.12 ^b^	0.67 ^m^	0.06 ^o^	0.25 ^de^	0.03 ^q^
UO4	10.13 ^c^	1.02 ^q^	2.51 ^a^	0.49 ^o^	0.22 ^m^	0.20 ^h^	0.24 ^h^
UO5	10.04 ^c^	2.77 ^d^	2.05 ^c^	0.36 ^q^	-	0.20 ^h^	0.08 ^m^
UO6	8.67 ^d^	2.85 ^d^	1.83 ^e^	-	-	0.22 ^g^	0.05 ^op^
UM1	10.32 ^b^	0.93 ^r^	1.19 ^i^	0.56 ^n^	2.03 ^f^	0.20 ^h^	0.37 ^e^
UM2	5.99 ^i^	2.59 ^e^	1.65 ^f^	1.72 ^c^	2.81 ^c^	0.23 ^fg^	0.48 ^b^
UM3	4.73 ^m^	2.27 ^g^	1.05 ^j^	1.06 ^h^	2.13 ^e^	0.28 ^c^	0.36 ^e^
UM4	3.80 ^q^	0.57 ^s^	0.68 ^p^	0.84 ^l^	2.14 ^e^	0.19 ^h^	0.43 ^d^
UM5	4.14 ^o^	1.82 ^m^	0.91 ^m^	1.21 ^f^	3.04 ^b^	0.24 ^ef^	0.62 ^a^
UM6	3.88 ^p^	1.54 ^o^	0.94 ^lm^	1.65 ^d^	2.36 ^d^	0.13 ^k^	0.46 ^c^
UR1	4.83 ^m^	1.51 ^p^	0.94 ^lm^	0.92 ^k^	-	0.12 ^l^	0.05 ^op^
UR2	5.88 ^j^	2.11 ^ij^	1.05 ^j^	0.79 ^l^	0.49 ^j^	0.16 ^i^	0.03 ^q^
UR3	5.29 ^k^	1.54 ^o^	1.07 ^j^	1.13 ^g^	0.71 ^i^	0.22 ^g^	0.04 ^p^
UR4	5.97 ^i^	1.55 ^o^	0.92 ^m^	2.05 ^a^	0.43 ^k^	0.10 ^m^	0.29 ^f^
UR5	3.67 ^r^	2.17 ^h^	0.96 ^l^	0.57 ^n^	-	0.22 ^g^	0.19 ^ij^
UR6	3.77 ^q^	2.03 ^k^	1.18 ^i^	0.57 ^n^	-	0.33 ^a^	0.17 ^k^
UR7	3.67 ^r^	1.69 ^n^	1.19 ^i^	1.01 ^i^	-	0.34 ^a^	0.26 ^g^
UJ1	13.70 ^a^	3.42 ^a^	1.90 ^d^	1.05 ^h^	0.69 ^i^	0.25 ^de^	0.12 ^l^
UJ2	7.54 ^e^	1.91 ^l^	1.15 ^i^	0.42 ^p^	-	0.20 ^h^	0.29 ^f^
UJ3	6.98 ^f^	1.50 ^p^	0.99 ^k^	1.53 ^e^	0.49 ^j^	0.11 ^l^	0.07 ^n^
UJ4	6.94 ^fg^	1.64 ^n^	0.71 ^p^	1.04 ^h^	0.90 ^h^	0.12 ^l^	0.04 ^p^
UE1	4.58 ^n^	2.60 ^e^	1.19 ^i^	0.97 ^j^	-	0.30 ^b^	0.18 ^jk^
TO1	3.54 ^s^	2.15 ^hi^	1.29 ^h^	0.31	-	0.23 ^fg^	0.20 ^i^
TM1	4.96 ^l^	2.08 ^j^	1.43 ^g^	1.76 ^c^	3.48 ^a^	0.25 ^de^	0.41 ^d^
TR1	2.72 ^u^	1.03 ^q^	0.50	1.00 ^i^	0.41 ^k^	0.12 ^l^	0.08 ^m^
TJ1	6.46 ^h^	1.76 ^m^	0.85 ^n^	1.85 ^b^	0.99 ^g^	0.16 ^i^	0.03 ^q^
TA1	3.19 ^t^	2.46 ^f^	0.70 ^p^	1.15 ^g^	0.36 ^l^	0.26 ^d^	0.06 ^o^
TV1	6.53 ^h^	3.31 ^b^	0.80 ^o^	0.97 ^j^	-	0.35 ^a^	0.20 ^i^

* Different letters within the same column indicate statistically significant differences at *p* < 0.05 by Tukey’s test.

**Table 5:** Amounts of individual polyphenols (mg kg^−1^ DW), Sum of quantified polyphenols (mg kg^−1^ DW), TPC (g GAE kg^−1^ DW), and RSA (mmol TE kg^−1^ DW) determined in the dried plum samples.

Sample ID	GalA	ChlA	Cat	CafA	Aes	Rut	CouA	Hyp	Iso-Rut	Iso-Glu	Kae-Glu	Que-Rha	EllA	Que	Iso	SUM	TPC	RSA
UO1	8.62 ^pq^*	196.03 ^g^	-	7.30 ^r^	-	13.98 ^n^	5.12 ^n^	2.39 ^n^	0.34 ^q^	0.08 ^q^	5.83 ^p^	0.13 ^o^	5.86 ^s^	1.68 ^t^	1.93 ^t^	249.29	5.94 ^q^	96.54 ^pq^
UO2	69.90 ^a^	269.96 ^d^	18.91 ^a^	21.31 ^d^	-	102.20 ^b^	-	32.82 ^a^	8.93 ^b^	5.62 ^a^	286.21 ^a^	5.88 ^a^	50.18 ^a^	3.00 ^n^	5.32 ^l^	880.24	6.83 ^o^	87.14 ^s^
UO3	10.34 ^m^	70.06 ^q^	-	11.58 ^m^	9.80 ^b^	8.66 ^s^	8.60 ^h^	4.60 ^hi^	0.92 ^j^	0.67 ^ef^	33.68 ^f^	0.91 ^e^	21.99 ^c^	2.34 ^p^	3.66 ^n^	187.81	8.93 ^h^	110.10 ^k^
UO4	18.00 ^f^	109.44 ^l^	7.71 ^e^	5.90 ^t^	-	12.34 ^q^	-	2.55 ^m^	0.50 ^o^	0.06 ^r^	7.66 ^o^	-	8.26 ^l^	4.59 ^j^	5.81 ^k^	182.82	14.95 ^c^	88.54 ^s^
UO5	21.49 ^e^	80.49 ^o^	16.54 ^b^	5.58 ^u^	-	24.51 ^i^	-	5.22 ^fg^	1.28 ^h^	0.45 ^ij^	21.35 ^i^	0.33 ^j^	8.84 ^i^	2.04 ^q^	3.61 ^n^	191.73	12.84 ^e^	101.77 ^n^
UO6	11.12 ^k^	68.45 ^q^	-	6.45 ^s^	-	16.87 ^m^	-	4.70 ^h^	1.67 ^f^	0.69 ^e^	31.69 ^g^	0.55 ^g^	11.35 ^f^	1.22 ^f^	1.89 ^u^	156.65	8.36 ^i^	95.44 ^q^
UM1	16.46 ^g^	86.27 ^n^	9.19 ^c^	10.46 ^o^	-	7.29 ^t^	-	1.34 ^r^	0.41 ^p^	0.03	3.13 ^t^	0.01 ^q^	8.70 j	7.75 ^g^	8.19 ^f^	159.23	15.72 ^b^	262.91 ^a^
UM2	-	250.17 ^e^	-	11.94 ^l^	-	77.86 ^c^	7.91 ^j^	8.65 ^b^	0.66 ^l^	0.58 ^g^	8.48 ^n^	1.86 ^b^	11.44 ^f^	15.40 ^c^	2.68 ^r^	397.63	8.15 ^j^	128.15 ^f^
UM3	-	309.74 ^c^	-	24.84 ^b^	-	25.28 ^hi^	20.07 ^d^	5.02 ^g^	0.43 ^p^	0.63 ^f^	8.20 ^n^	1.89 ^b^	7.37 ^m^	5.53 ^i^	1.11 ^w^	410.11	14.17 ^d^	100.32 ^o^
UM4	10.67 ^l^	6.88 ^w^	-	3.98 ^x^	5.65 ^e^	1.60 ^x^	-	1.23 ^s^	-	0.08 ^q^	5.67 ^q^	0.04 ^p^	6.24 ^q^	1.82 ^rs^	1.46 ^v^	45.32	4.43 ^u^	123.86 ^g^
UM5	8.86 ^p^	129.32 ^j^	-	14.02 ^i^	4.79 ^h^	38.63 ^f^	5.19 ^n^	4.78 ^h^	0.77 ^k^	0.22 ^mn^	17.14 ^k^	0.29 ^k^	9.33 ^h^	11.70 ^d^	6.95 ^h^	251.99	8.02 ^k^	105.08 ^m^
UM6	8.39 ^q^	165.54 ^i^	-	14.84 ^h^	5.71 ^e^	20.27 ^k^	10.05 ^f^	3.68 ^k^	0.36 ^q^	0.23 ^lm^	21.30 ^i^	0.37 ^i^	8.71 ^j^	6.26 ^h^	3.05 ^p^	268.76	7.42 ^n^	35.42 ^v^
UR1	14.88 ^h^	95.01 ^m^	-	12.98 ^j^	3.45 ^j^	10.83 ^p^	-	2.92 ^l^	0.67 ^l^	0.44 ^j^	19.32 ^j^	0.37 ^i^	6.29 ^q^	3.92 ^k^	9.15 ^e^	180.23	5.93 ^q^	128.16 ^f^
UR2	9.32 ^o^	141.07 ^j^	8.11 ^d^	15.27 ^g^	10.28 ^a^	15.48 ^n^	9.37 ^g^	5.48 ^ef^	1.05 ^i^	0.78 ^d^	39.86 ^d^	0.46 ^h^	9.36 ^h^	3.46 ^l^	6.27 ^i^	275.62	4.68 ^t^	100.13 ^o^
UR3	10.36 ^m^	76.13 ^p^	-	12.60 ^k^	-	13.55 ^o^	9.28 ^g^	4.50 ^i^	0.90 ^j^	0.71 ^e^	37.06 ^e^	0.60 ^f^	8.47 ^k^	3.11 ^mn^	6.88 ^h^	184.15	5.16 ^s^	105.60 ^m^
UR4	9.42 ^no^	28.31 ^u^	-	7.13 ^r^	-	1.58 ^x^	-	0.80 ^u^	0.04 ^t^	-	0.76 ^w^	-	6.23 ^q^	1.70 ^t^	6.06 ^j^	62.03	7.35 ^n^	141.72 ^c^
UR5	11.86 ^jk^	14.20 ^v^	-	4.92 ^w^	4.93 ^g^	3.29 ^w^	8.20 ^i^	1.79 ^p^	0.06 ^s^	0.13 ^p^	8.43 ^n^	0.13 ^o^	6.65 ^o^	1.90 ^r^	2.94 ^p^	69.43	5.79 ^r^	119.52 ^h^
UR6	0.23 ^v^	172.41 ^h^	-	17.10 ^f^	6.68 ^d^	13.40 ^o^	21.48 ^c^	1.13 ^t^	0.41 ^p^	0.17 ^o^	3.54 ^s^	0.25 ^l^	-	1.76 ^st^	2.78 ^q^	241.34	9.32 ^g^	74.49 ^t^
UR7	6.38 ^s^	172.22 ^h^	-	14.27	5.47 ^f^	22.02 ^j^	21.85 ^c^	1.97 ^o^	1.47 ^g^	0.23 ^lm^	5.14 ^r^	0.26 ^l^	-	2.04 ^q^	7.16 ^g^	260.48	8.01 ^k^	74.58 ^t^
UJ1	22.74 ^e^	335.49 ^b^	7.74 ^e^	41.82 ^a^	6.73 ^d^	49.91 ^d^	24.96 ^a^	7.87 ^c^	12.32 ^a^	1.10 ^c^	9.41 ^n^	0.36 ^ij^	16.87 ^d^	33.17 ^a^	86.89 ^a^	657.38	16.24 ^a^	208.99 ^b^
UJ2	13.26 ^i^	225.33 ^f^	-	18.07 ^e^	-	43.96 ^e^	17.48 ^e^	7.01 ^d^	6.44 ^c^	1.48 ^b^	49.02 ^c^	0.96 ^d^	13.81 ^e^	9.82 ^e^	25.02 ^b^	431.66	8.92 ^h^	136.69 ^d^
UJ3	7.63 ^r^	59.39 ^r^	-	11.31 ^n^	-	6.39 ^u^	9.99 ^f^	1.15 ^t^	1.32 ^h^	0.09 ^q^	2.07 ^v^	-	6.89 ^n^	2.78 ^o^	9.25 ^e^	118.26	5.80 ^r^	115.48 ^i^
UJ4	2.48 ^t^	86.29 ^n^	-	14.56 ^hi^	-	12.85 ^p^	7.00 ^l^	1.57 ^q^	1.93 ^e^	0.14 ^p^	2.75 ^u^	0.13 ^o^	6.61 ^o^	3.33 ^lm^	10.39 ^d^	150.03	6.11 ^p^	123.89 ^g^
UE1	12.43 ^j^	67.91 ^q^	-	8.14 ^q^	5.64 ^e^	26.21 ^h^	-	7.96 ^c^	1.82 ^e^	1.10 ^c^	63.15 ^b^	1.06 ^c^	14.21 ^e^	6.01 ^h^	5.91 ^k^	221.55	11.69 ^f^	112.69 ^j^
TO1	48.82 ^b^	263.11 ^d^	7.13 ^f^	9.32 ^p^	5.77 ^e^	32.49 ^g^	-	5.69 ^e^	1.32 ^h^	0.32 ^k^	18.24 ^jk^	0.34 ^j^	9.52 ^g^	1.32 ^u^	2.09 ^s^	405.48	7.13 ^o^	108.57 ^l^
TM1	34.53 ^c^	57.96 ^r^	-	7.95 ^q^	5.51 ^f^	18.01 ^l^	5.56 ^m^	1.98 ^o^	0.12 ^r^	0.16 ^o^	5.13 ^r^	0.17 ^m^	7.76 ^l^	8.35 ^f^	6.32 ^i^	159.51	9.06 ^h^	130.54 ^e^
TR1	9.68 ^n^	29.98 ^t^	6.85 ^f^	5.25 ^v^	4.01 ^i^	5.95 ^v^	-	2.31 ^n^	0.57 ^n^	0.24 ^l^	15.52 ^l^	-	6.51 ^p^	2.08 ^q^	3.36 ^o^	92.31	7.56 ^m^	87.94 s
TJ1	10.23 ^m^	121.55 ^k^	-	12.87 ^jk^	7.32 ^c^	13.65 ^no^	7.54 ^k^	3.77 ^k^	2.78 ^d^	0.48 ^i^	24.79 ^h^	0.44 ^h^	6.51 ^p^	4.50 ^j^	13.87 ^c^	230.30	5.85 ^r^	90.23 ^r^
TA1	29.99 ^d^	52.01 ^s^	-	11.55 ^mn^	-	9.56 ^r^	5.41 ^m^	3.93 ^j^	0.60 ^m^	0.54 ^h^	25.43 ^h^	0.56 ^g^	6.12 ^r^	1.70 ^t^	2.86 ^pq^	150.26	7.79 ^l^	97.13 ^p^
TV1	0.56 ^u^	503.90 ^a^	-	34.51 ^b^	-	128.26 ^a^	23.52 ^b^	8.61 ^b^	0.59 ^mn^	0.21 ^n^	14.01 ^m^	0.15 ^n^	25.48 ^b^	19.41 ^b^	4.08 ^m^	763.29	11.50 ^f^	63.05 ^u^
Average	14.62	141.49	2.74	13.26	3.06	25.90	7.62	4.91	1.69	0.59	26.47	0.62	10.52	5.79	8.56		8.66	111.82

GalA—Gallic acid; ChlA—Chlorogenic acid; Cat—Catechin; CafA—Caffeic acid; Aes—Aesculin; Rut—Rutin; CouA—p-Coumaric acid; Hyp—Hyperoside; Iso-Rut—Isorhamnetin -3-O-rutinoside; Iso-Glu—Isorhamnetin -3-O-glucoside; Kae-Glu—Kaempferol-3-O-glucoside; Que-Rha—Quercetin-3-O-rhamnoside; EllA—Ellagic acid; Que—Quercetin; Iso—Isorhamnetin; SUM–The total sum of sugars. * Different letters within the same column indicate statistically significant differences at *p* < 0.05 by Tukey’s test.

## Data Availability

Data are contained within the article.

## Supplementary Materials

The following supporting information can be downloaded at: https://www.mdpi.com/article/10.3390/antiox13050526/s1, Figure S1: Principal component analysis performed on TPC, RSA, and individual polyphenols (A and B); elemental composition (C and D); and sugar and sugar alcohol contents (E and F). Abbreviations in Figure S1B correspond to polyphenols presented in Table 5.
