# Supplementary figures and images for: Profiling of Metabolites in Organically Grown Plums from Norway: Does Location or Cultivar Matter?
Source: Antioxidants (Basel). 2024 Apr 26;13(5):526. doi: 10.3390/antiox13050526 (PMC11117866; doi:10.3390/antiox13050526)

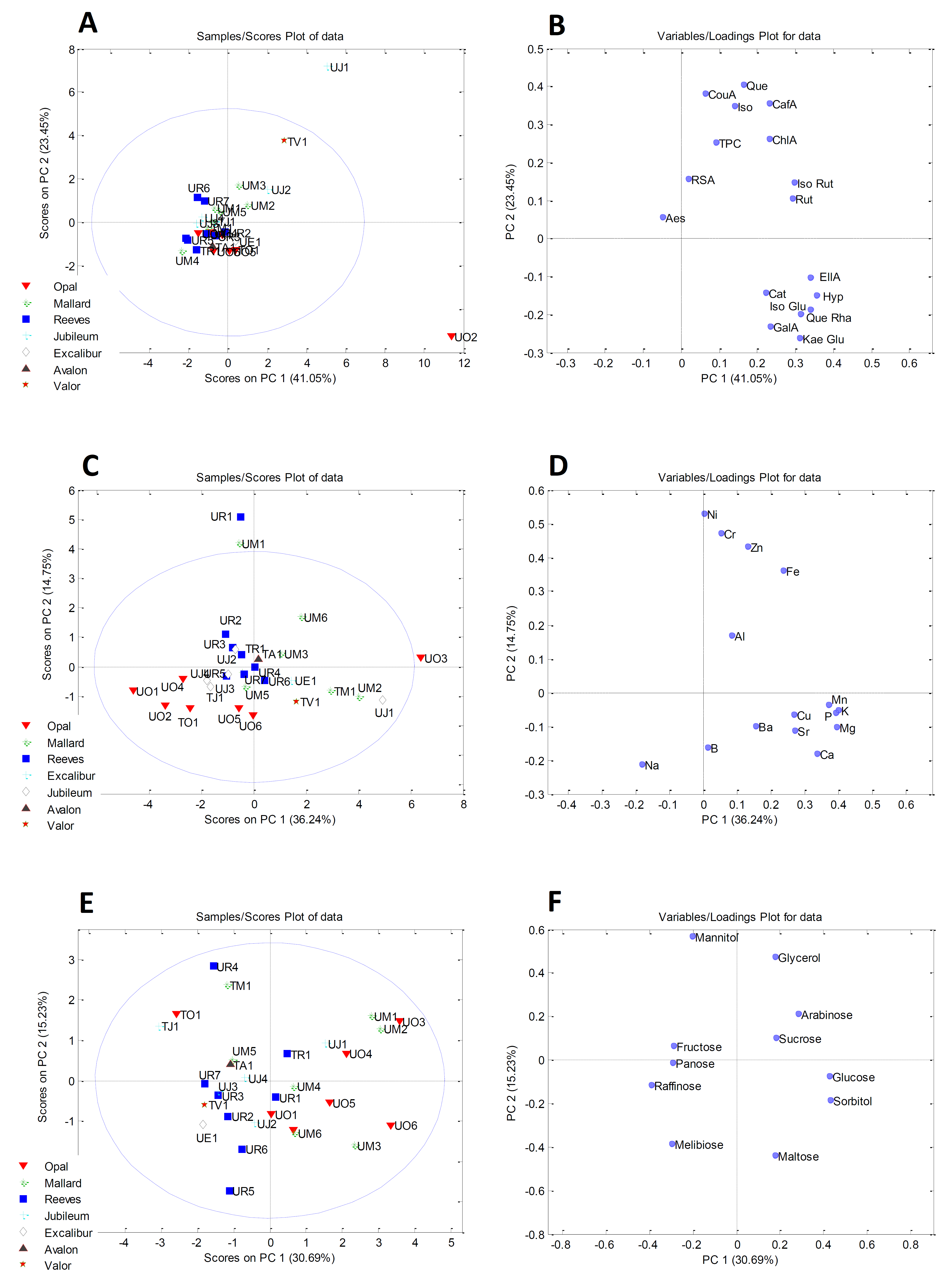

Supplement: Supplementary file 1 [file antioxidants-13-00526-s001.zip › antioxidants-2970348-supplementary.tif]
